# Pedunculated Angiomyofibroblastoma of the Vulva: Case Report and Review of the Literature

**DOI:** 10.1155/2011/893261

**Published:** 2011-09-15

**Authors:** Luca Giannella, Matteo Costantini, Kabala Mfuta, Alberto Cavazza, Lillo Bruno Cerami, Giorgio Gardini, Fausto Boselli

**Affiliations:** ^1^Division of Obstetrics and Gynaecology, Cesare Magati Hospital, Scandiano, 42019 Reggio Emilia, Italy; ^2^Department of Pathology, Santa Maria Nuova Hospital, 42123 Reggio Emilia, Italy; ^3^Oncology Unit, Division of Obstetrics and Gynecology, University of Modena and Reggio Emilia, 41124 Modena, Italy

## Abstract

Angiomyofibroblastoma (AMFB) is a rare benign mesenchymal tumour that occurs almost exclusively in the vulvovaginal region of women but can also occur occasionally in the inguinoscrotal region of men. It is a well-circumscribed lesion that clinically is often thought to represent a Bartholin's gland cyst and usually does not form a pedunculated mass. To our knowledge, only five cases of vulvar AMFB with pedunculated mass have been reported in the English literature and all cases involving the labia majora and middle-aged women. We report the first case of pedunculated AMFB of the vulva occurring in a young woman of 21 years old and involving the left labia minora. After excluding the most common diseases, pedunculated AMFB should be part of differential diagnosis in the workup of any pedunculated vulvar mass even in young women with a lesion involving the labia minora. We reviewed the literature and summarized all reported cases.

## 1. Introduction

Angiomyofibroblastoma (AMFB) is a rare benign mesenchymal tumor usually occurring in the vulvovaginal area of middle-aged women [1–3]. Histologically, the tumor is a well-circumscribed lesion composed of alternating hypo- and hypercellular areas with numerous delicate capillary-sized vessels [4]. AMFB can be distinguished from the aggressive angiomyxoma by its circumscribed borders, the presence of plump stromal cells, and perivascular condensation of the stromal cells [5]. Immunoreactivity for both desmin and vimentin is detected in almost all tumor cells, which also reveal estrogen and progesterone receptors [6]. Clinically, AMFB shows a slowly growing mass which is often misdiagnosed as a Bartholin's gland cyst and usually does not form a pedunculated mass [7]. To our knowledge, only five cases of vulvar AMFB with pedunculated mass have been reported in the English literature and all cases involving the labia majora and middle-aged women [1, 7–10]. We report the first case of pedunculated AMFB of the vulva occurring in a young woman of 21 years old and involving the left labia minora. 

## 2. Case Presentation

**Figure 1:**
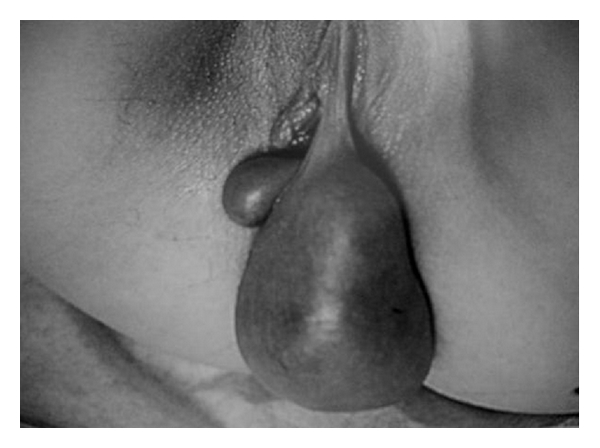
The tumor shows a pedunculated large mass arising from the labia minora with a subtle stalk.

A 21-year-old woman came to our observation reporting the presence of a painless vulvar neoformation starting four years before, which had grown during the last months. She ignored the mass, despite its gradual enlargement during her disease course, and the complaint was of discomfort when seated and a localized burning sensation. Her personal history was noncontributory for previous surgery and laboratory data (emocrome, transaminase, sideremia, ferritin, creatinine, coagulation) showed no significant abnormalities. Clinical examination showed a pedunculated bilobulated neoformation of ten centimeters in diameter of tense elastic consistency, at the level of the left labia minora (Figure 1). On gynecological examination and vaginal ultrasound, the uterus and bilateral uterine adnexae showed no abnormalities. Inguinal lymph nodes were not swollen and tumor markers (cancer antigen (CA) 125, CA-19.9, CA-15.3, Alphafetoprotein and carcinoembryonic antigen) were negative. These markers were performed to exclude a concomitant malignancy in other locations. On ultrasound examination the mass revealed a well-demarcated soft tissue tumor with homogeneous echo and normal vascularity. In order to exclude a surgical disease, such as perineal hernia [11–13], the patient was evaluated by surgeons that, according with personal history, clinical examination, and ultrasonography, excluded a lesion of their interest.

The mass gave a clinical impression of vulvar fibroid, and a simple tumor excision was subsequently performed at the site of the stalk. During surgery, to exclude a malignancy lesion, a rapid intraoperative pathologic diagnosis of the tumor was performed, and it was reported as a benign soft lesion of the vulva. On gross examination, the lesion was well circumscribed, weighed 1230 gr, had a soft to firm consistency, and appeared homogeneously light gray to tan in color on the cut surface. No foci of hemorrhage nor necrosis was detected.

**Figure 2:**
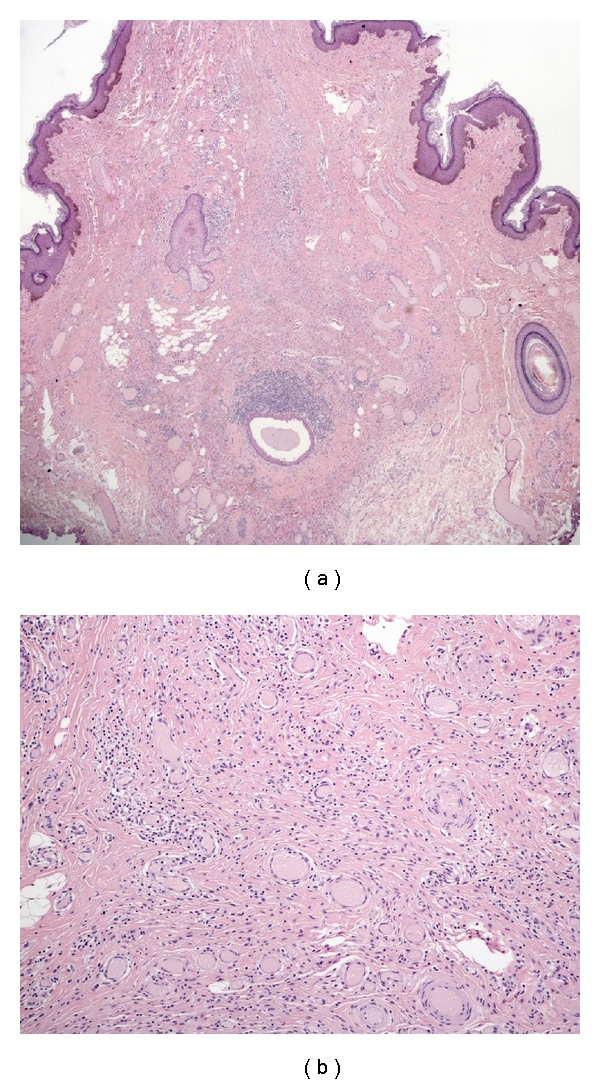
(a) Photomicrograph (hematoxylin-eosin, original magnification ×2) showing pedunculated lesion covered with skin. Note the proliferation of mesenchymal cells in richly vascularized fibrous stroma. (b) Photomicrograph (hematoxylin-eosin, original magnification ×10) showing a mixture of hypercellular and hypocellular edematous areas with abundant small- to medium-sized vessels.

**Figure 3:**
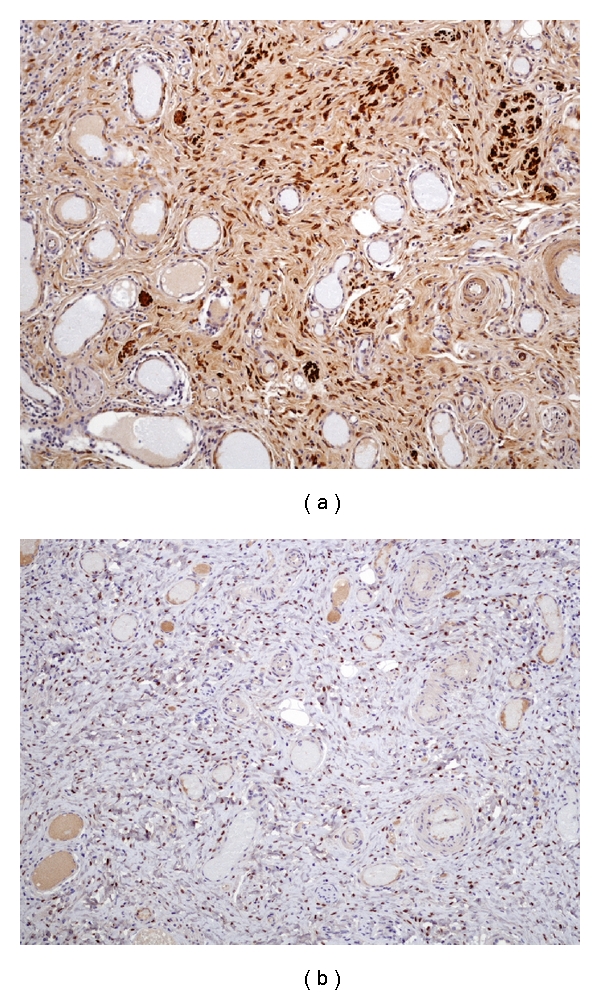
(a) Photomicrograph (immunohistochemical staining for desmin, original magnification ×10) showing desmin positivity for mesenchymal proliferation. (b) Photomicrograph (immunohistochemical staining for estrogen receptors, original magnification ×10) showing positivity of stromal elements for estrogen receptors.

Microscopically, the mass consisted of fibroconnective tissue with abundant vessels of various wall thickness (Figures 2(a) and 2(b)). No mitotic or atypical cells were seen, and the stroma was edematous. In immunohistochemistry, tumor cells were strongly positive for desmin and estrogen receptors (Figures 3(a) and 3(b)) and negative for *α*-smooth muscle actin, S-100 protein, cytokeratin, and CD34. All these features were in favour of the diagnosis of AMFB. Surgical margins of the lesion were clear, and the pedicle was not involved by the tumor.

Currently the patient is in followup for two years with clinical examination every six months with no evidence of recurrence.

## 3. Discussion

This distinctive tumor was first delineated by Fletcher et al. in 1992 who described 10 cases involving the vulva [8] with only one case reporting a large pedunculated mass. There is a marked predilection for the female genital tract, predominantly the vulva, although rare cases have also been reported to arise in the scrotum and the inguinal area in males [8, 14]. The female patients have an average age of 45.8 years [5]. They usually complain of a painless mass that has been present from a few weeks to up to 13 years [5]. Clinically, these tumors are frequently thought to be a Bartholin gland cyst, although a preoperative differential diagnosis includes labial cysts, inguinal hernia, leiomyoma, and mesenchymal tumors such as lipoma and liposarcoma [15]. 

On gross examination they are typically well circumscribed and ranged from 0.5 to 12 cm. They have a soft to rubbery consistency and a bulging, pink, and somewhat lobulated sectioned surface [5]. Microscopic examination confirms the well-demarcated nature of the lesion and shows alternating hypercellular and hypocellular edematous regions with abundant blood vessels. There is minimal nuclear atypicality, and mitotic figures are rare. The cells tend to cluster around blood vessels, sometime forming compact foci [5]. Adipocytes can be sparsely scattered within the neoplasm and in rare cases fat predominates; these tumors have been classified as the “lipomatous” variant of angiomyofibroblastoma [16]. The angiomyofibroblastoma can be distinguished from the aggressive angiomyxoma by its circumscribed borders, the presence of plump stromal cells that are occasionally overtly epithelioid, and perivascular condensation of the stromal cells [17]. A single case of a malignant transformation of an AMFB in angiomyofibrosarcoma has been reported [18]. In that tumor, areas of typical AMFB merged imperceptibly with high-grade sarcoma resembling a myxoid malignant fibrous histiocytoma. 

Immunohistochemical stains show uniform staining for vimentin and staining of a variable number of cells for desmin in most cases. The tumor cells also show variable expression for muscle actin and smooth muscle actin and are frequently positive for estrogen and progesterone receptors [5]. The expression of estrogen and progesterone receptors suggests that it might arise as a neoplastic proliferation of hormonally responsible mesenchymal cells [3]. Some tumors have also stained for CD34, although some authors question whether these CD34 positive tumors might present fibroepithelial polyps, which are frequently positive for this marker [16]. 

The tumor usually exists as a sharply circumscribed mass in the subcutaneous tissue of the vulva and usually does not form a pedunculated mass, which represents an exceptional event [7]. To our knowledge, only five cases of pedunculated AMFB of the vulva have been reported in the English literature [1, 7–10]. 

All cases are reported in Table 1. All patients underwent simple excision. The mean age of patients was 46.4 years with a range of 41–50 years. The lesion always involved the labia majora. The average size of the pedunculated AMFB was of 14.2 × 13.6 centimeters in diameter (range 4 to 23 cm). Except for one case, the presence of the lesion had existed for several years. The real problem is the preoperative clinical diagnosis, as the vulvar AMFB is a rare event revealed only by histological examination after surgery. All patients had an uneventful postoperative course, and the mean followup was of 20 months (ranging from 8 months to 3 years) with no recurrence. 

Table 2 shows immunohistochemical staining for the most common antibodies in the AMFB. Almost all tumor cells were strongly positive for vimentin and desmin, but uniformly negative for cytokeratin and S-100 protein. Not all tumor cells were negative for *α*-smooth muscle actin and CD34. Staining for estrogen and progesterone receptors was observed in the nucleus of all tumor cells. 

Given the data reported in the literature on the pedunculated AMFB of the vulva, our case is the first concerning a young woman of 21 years old and involving the labia minora. The patient underwent simple excision with clear margin of the lesion and currently is in followup performing clinical examination every six months for two years with no recurrence. 

This paper aims to emphasize that, after excluding the most common vulvar diseases, the appearance of a pedunculated AMFB should be part of differential diagnosis in the workup of any pedunculated vulvar mass even in young women with a lesion involving the labia minora. The treatment of choice is a surgical excision with clear margins, which is resolutive as demonstrated by reported cases in the literature. 

## Figures and Tables

**Table 1:** Clinical features of pedunculated AMFB of the vulva.

References	Patient no.	Age (years)	Site	size (cm)	Duration	Clinical diagnosis	Outcome
Fletcher et al. [8]	1	41	Right labia majora	4 × 12	8 years	Inguinal hernia	No recurrence after 2 years
Hsu et al. [1]	2	45	Left labia majora	13 × 12	6 months	Not specified	No recurrence after 8 months
Omori et al. [7]	3	48	Left labia majora	11 × 9	7 years	Lipoma	No recurrence after 3 years
Barat et al. [9]	4	50	Left labia majora	20 × 15	6 years	Ulcerated vulvar mass	No recurrence after 8 months
Nagai et al. [10]	5	48	Right labia majora	23 × 20	3 years	Not specified	No recurrence after 2 years

**Table 2:** Immunohistochemical staining for the most common antibodies in the AMFB.

Patient no.	Desmin	Vimentin	e-R	p-R	S-100 p	CD34	ASMA	Cytokeratin
1	+	+	U	U	−	U	−	−
2	+	U	+	+	−	−	±	U
3	±	+	+	+	−	+	±	−
4	+	+	+	+	−	U	U	−
5	+	+	+	+	−	−	−	U

+ = positive; − = negative; ± = low positive; U = unchecked; e-R = estrogen receptor; p-R = progesterone receptor; S-100 p = S-100 protein; ASMA = *α*-smooth muscle actin.
